# DAXX inhibits cancer stemness and epithelial–mesenchymal transition in gastric cancer

**DOI:** 10.1038/s41416-020-0800-3

**Published:** 2020-03-23

**Authors:** Chaofan Wu, Hui Ding, Shuochen Wang, Yangxin Li, Song-Bai Liu, Xiaoxiao Wang, Jiqing Zheng, Ting Xue, Hesham M. Amin, Yao-Hua Song, Jin Zhou

**Affiliations:** 1 Cyrus Tang Hematology Center, Collaborative Innovation Center of Hematology, Soochow University, Suzhou, P. R. China; 2 Department of Cardiovascular Surgery & Institute of Cardiovascular Science, First Affiliated Hospital of Soochow University, 215123 Suzhou, Jiangsu P. R. China; 3 Suzhou Vocational Health College, Suzhou Key Laboratory of Biotechnology for Laboratory Medicine, Suzhou, 215009 Jiangsu Province China; 4 Department of Hematopathology, The University of Texas MD Anderson Cancer Center, 1515 Holcombe Boulevard, Houston, TX USA; 5 Department of General Surgery, the First Affiliated Hospital of Soochow University, Suzhou, P. R. China

**Keywords:** Gastric cancer, Gastrointestinal cancer

## Abstract

**Background:**

DAXX is a transcription repressor that has been implicated in several types of cancers, but its role in the development of gastric cancer remains unknown.

**Methods:**

We analysed the expression of DAXX in 83 pairs of gastric cancer samples, including neoplastic and adjacent tissues, and correlated the expression levels with clinical stages. We also investigated the molecular mechanisms by which DAXX downregulation promotes cancer growth using both in vitro and in vivo models.

**Results:**

DAXX was downregulated in advanced gastric cancer samples. The expression of DAXX inversely correlates with that of cancer stem cell markers CD44 and Oct4 in gastric cancer lines. DAXX overexpression in gastric cancer cells inhibited migration, invasion and epithelial– mesenchymal transition (EMT). The inhibition of EMT was achieved through the repression of SNAI3, a key inducer of EMT, by recruiting HDAC-1 into the nucleus. Using a xenograft mouse model, we demonstrated that the MKN45 cells formed smaller tumours when DAXX was overexpressed. Wild-type AGS cells were not able to form tumours in nude mice, but in contrast, formed visible tumours when DAXX was silenced in the cells.

**Conclusion:**

We for the first time demonstrated that DAXX functions as a tumour suppressor in gastric cancer by inhibiting stem cell growth and EMT.

## Background

Death domain-associated protein (DAXX) is a transcription repressor involved in both physiological and pathological conditions. The role of DAXX in the development of cancer remains controversial. It has been shown that DAXX expression is upregulated in gliomas and ovarian cancer.^1,2^ It was also shown that DAXX promotes prostate cancer tumorigenicity by repressing autophagy machinery.^3^ In contrast, loss of DAXX is associated with reduced survival in patients with pancreatic neuroendocrine tumours.^4^ In cooperation with menin, DAXX inhibits the proliferation of insulinoma cells through epigenetic regulation of membrane metalloendopeptidase.^5^ In breast cancer, DAXX is a potent inhibitor of breast tumour-initiating cells (TIC), and that DAXX represses pluripotent and EMT gene expression by potentially binding promoters of pluripotent TIC-associated genes.^6^ Thus, it is believed that DAXX can either promote or inhibit the development of malignant neoplasms, depending on cell type and context.

Cancer stem cells and epithelial–mesenchymal transition (EMT) play important roles in tumour growth and metastasis. Previous studies showed that high expression of CD44 and Oct4 is associated with poor survival.^7,8^ However, the involvement of DAXX in regulating the expression of CD44 and Oct4 has not been reported. A recent study showed that DAXX suppresses lung cancer metastasis by inhibiting EMT.^9^ The role of DAXX in EMT in other types of cancers, including gastric cancer, remains unknown. In this study, we showed for the first time that DAXX inhibits the development of gastric cancer by inhibiting SNAI3- mediated EMT. We further showed that knockdown of DAXX in gastric cancer cells sustains cell survival, enhances the expression of stem cell markers CD44 and Oct4 and promotes tumour growth.

## Methods

### Cell culture

Human gastric carcinoma cell line MKN45 was purchased from China Infrastructure of Cell Line Resources (Beijing, China), and cultured in RPMI supplemented with 20% foetal bovine serum. AGS and N87 cells were purchased from Cell Bank of the Chinese Academy of Sciences (Shanghai, China). AGS cells were cultured in Ham’s F-12K containing 10% foetal bovine serum. The N87 cells were cultured in RPMI 1640 supplemented with 10% foetal bovine serum. The identities of all cell lines used in this study were recently verified by short tandem repeat profiling analysis. The cell lines have recently been tested for mycoplasma contamination.

### Western blot

The procedures for Western blot have been described previously.^10^ The mouse anti-E-cadherin (1/1000), rabbit anti-DAXX (1/1000), vimentin (1/500), GAPDH (1/5000) and HDAC-1 (1/2000) were from Cell Signaling Technologies (Danvers, MA). The rabbit anti-histone H3 (acetyl K14) (1/2000), histone H3 (acetyl K27) (1/1000), histone H3 (acetyl K9) (1/10000), histone H3 (acetyl K18) (1/1000) and histone H3 (acetyl K23) (1/1000) were from Abcam. Horseradish peroxidase-conjugated secondary antibodies (anti-rabbit IgG: 1:5000, or anti-mouse IgG: 1:5000) were from Sigma (St. Louis, MO).

### Co-immunoprecipitation

The cell lysates were incubated with a rabbit anti-HA-tag antibody (1/50, Cell Signaling), or anti-HDAC-1 antibody (1/100, Cell Signaling), or anti-DAXX antibody (1/50, Cell Signaling) at 4 °C overnight. Isotype-matched IgG was used as control. Protein A/G agarose was added to the solution and incubated for 3 h. Finally, the precipitated proteins were analysed by Western blot to detect the expression of DAXX and HDAC-1. The rabbit anti-DAXX (1/1000) and HDAC-1 (1/2000) were from cell signaling.

### Immunofluorescence staining

Cells were fixed in 4% paraformaldehyde, and immunofluorescence staining was performed as described previously.^11^ Antibodies against DAXX, E-cadherin, vimentin, CD44 and HDAC-1 were purchased from Cell Signaling (1/100). Antibodies against CD24 were purchased from Abcam (1/100). Goat anti-rabbit IgG—Alexa Fluor^®^ 568 conjugate and goat anti-mouse IgG—Alexa Fluor^®^488 conjugate were purchased from Thermo Fisher Scientific (Waltham, MA). DAPI was added at the final step to reveal nuclei.

### Flow cytometry analysis

Gastric cancer cell lines were incubated with PE-labelled mouse anti-human CD44 antibody (BD Biosciences, 550989), and PerCP-Cy5.5-labelled mouse anti-human CD24 antibody (BD Biosciences, 561647) for 30 min at 4 °C, and analysed using a flow cytometer (FACS Calibur, Becton Dickinson). The data were analysed using FlowJo software.

### Immunohistochemistry

The sections were incubated with the primary antibody mouse anti-CD44 (Cell Signaling Technology) at 4 °C overnight. The sections were incubated with GTVision^TM^ III peroxidase-labelled secondary antibody (Gene Tech, GK500705) in blocking solution at 37 °C for 45 min. After three washes in PBS, the sections were incubated in DAB working solution for 2 min.

### Cell viability assay

Cell viability was analysed using CCK8 reagent in 96-well plates as described previously.^10^

### Apoptosis

Apoptosis was detected by flow cytometry using Annexin V and propidium iodide (PI) staining kit (BD Pharmingen, Bedford, MA).^12^

### Real-time PCR

RT-PCR was performed as described previously,^11^ using the ABI 7500 Real-time PCR System with the following primers:

DAXX-F: GAAGCCTCCTTGGATTCTGGTG

DAXX-R: CATCACTCTCCTCATCGTCTTCG

CD44-F: CTGCCGCTTTGCAGGTGTA

CD44-R: CATTGTGGGCAAGGTGCTATT

OCT-4-F: CTTGAATCCCGAATGGAAAGGG

OCT-4-R: GTGTATATCCCAGGGTGATCCTC

GAPDH-F: ACCCAGAAGACTGTGGATGG

GAPDH-R: CAGTGAGCTTCCCGTTCAG

cIAP1-F: CAGACACATGCAGCTCGAATGAG

cIAP1-R: CACCTCAAGCCACCATCACAAC

cIAP2-F: GCTTTTGCTGTGATGGTGGACTC

cIAP2-R: CTTGACGGATGAACTCCTGTCC

Survivin-F: CCACTGAGAACGAGCCAGACTT

Survivin-R: GTATTACAGGCGTAAGCCACCG

XIAP-F: TGGCAGATTATGAAGCACGGATC

XIAP-R: AGTTAGCCCTCCTCCACAGTGA

Bcl-XL-F: GCCACTTACCTGAATGACCACC

Bcl-XL-R: AACCAGCGGTTGAAGCGTTCCT

cFLIP-F: AGTGAGGCGATTTGACCTGCTC

cFLIP-R: CCTCACCAATCTCTGCCATCAG

SNAI1-F: TGCCCTCAAGATGCACATCCGA

SNAI1-R: GGGACAGGAGAAGGGCTTCTC

SNAI2-F: ATCTGCGGCAAGGCGTTTTCCA

SNAI2-R: GAGCCCTCAGATTTGACCTGTC

SNAI3-F: TGCACCTGCAAGATCTGTGGCA

SNAI3-R: AAGGTTGGAGCGGTCGGCAAAG

ZEB1-F: GGCATACACCTACTCAACTACGG

ZEB1-R: TGGGCGGTGTAGAATCAGAGTC

GRHL2-F: CGCCTATCTCAAAGACGACCAG

GRHL2-R: CCAGGGTGTACTGAAATGTGCC

OVOL2-F: CCACAACCAGGTGAAAAGACACC

OVOL2-R: CGCTGGGTGAAGGCTTTATTGC

E-Cadherin-F: GCCTCCTGAAAAGAGAGTGGAAG

E-Cadherin-R: TGGCAGTGTCTCTCCAAATCCG

Vimentin-F: AGGCAAAGCAGGAGTCCACTGA

Vimentin-R: ATCTGGCGTTCCAGGGACTCAT

CD24-F: CACGCAGATTTATTCCAGTGAAAC

CD24-R: GACCACGAAGAGACTGGCTGTT

### Wound-healing assay

Cells were plated in six-well plates and cultured overnight, then wounded with a 200-µL pipette tip as described previously.^13^

### Migration and invasion assays

Cell migration and invasion were performed in 24-well Transwell units (Costar, Lowell, MA) as described previously by our group.^13^

### Soft agar colony-formation assay

The 35-mm cell culture dishes (Corning, Lowell, MA) were first coated with 0.5% agar (1 ml), then 5 × 10^3^ cells suspended in 1 ml of 0.4% agar containing cell culture medium and 10% foetal bovine serum was added on top of the base layer.^14^ Colonies were photographed after 10–25 days using Olympus SZX16 microscope.

### Xenograft mouse model

MKN45 cells (8 × 10^6^) or AGS cells (1 × 10^7^) in 100 µl of PBS solution were mixed with 100 µl of Matrigel, and then injected subcutaneously into the back region of nude mice. Tumour volume was calculated using the formula *V* = 0.52 × length × width^2^ as described previously.^15^

### Experimental animals

Animal protocols were approved by the Institutional Laboratory Animal Care and Use Committee of Soochow University. All animal experiments complied with the arrive guidelines, and were carried out in accordance with the National Institutes of Health guidelines for the care and use of laboratory animals (NIH Publications No. 8023, revised 1978). Six-week-old male nude mice (Shanghai SLAC Laboratory Animal Co., Ltd) with an average body weight of 24 g were maintained in a special pathogen-free animal facility at Soochow University.

### Animal housing

Animals were housed in SPF facility. Nude mice were kept in individually ventilated cages (IVC), Euro Standard Type IIL in groups of six with filter tops (both Techniplast, Germany). Temperature in animal facilities was 22–26 °C, humidity was 55 ± 10%. Light cycle was 12 h. Housing was enriched by nesting material, plastic houses as well as wooden sticks; food and water were available ad libitum. Well-being of the animals was monitored daily.

### Sample size

A total of 30 6-week-old nude mice were used in the xenograft mouse model experiment, and each group of six was divided into a group of five groups.

### Experimental procedures

Five groups of mice were injected with different cells. The two groups of mice with DAXX overexpression experiments were injected with MKN45-LV5 and MKN45-DAXX, respectively. The three groups of DAXX-knockdown experiments were injected with AGS-NC, AGS-1044 and AGS-1503. MKN45 cells (8 × 10^6^) or AGS cells (1 × 10^7^) in 100 µl of PBS solution were mixed with 100 µl of Matrigel, and then injected subcutaneously into the back region of nude mice.

The overexpression experiment lasted for 21 days, and knockdown experiment lasted for 35 days. After the mouse was euthanised by cervical dislocation, the tumour was taken out from the skin of the mouse, and the volume was measured and weighed.

### Chromatin immunoprecipitation

The ChIP assay was carried out using the Chip-IT Express KIT (Active motif). Cells were cross-linked with 1% formaldehyde for 10 min, and then lysed in lysis buffer for 30 min. Nuclei were separated by centrifugation. DNA samples were sonicated to fragment sizes of 200–500 bp. After pre-clearing with normal IgG, the anti-histone H3 (acetyl K14) antibody (Abcam, 4 µl per 1 × 10^7^ cells) and protein G Magnetic beads were added and incubated at 4 °C overnight. After washing, the cross-link was reversed, and DNA was purified. The enrichment of SNAI3 was detected with the following primers:

Primer1-F: AGGAACGGAAGACACGGAAAGG

Primer1-R/ Primer2-F: CTGAGTAACACCAGGCTCTAGC

Primer2-R/ Primer3-F: AGGAGAGGCGGGCACCTTCTT

Primer3-R/ Primer4-F: ACTGAAGATGGGTGTTGCCACG

Primer4-R/ Primer5-F: CAGAGCTTCCATGGACTCACCA

Primer5-R/ Primer6-F: CAGGTCACACGTGCAGCTGTT

Primer6-R/ Primer7-F: CCCCATCAATCCTGTCCTGTGA

Primer7-R/ Primer8-F: AGAGAATTCCCGTGGGGAGGA

Primer8-R: CCGCGCTCCTTCCTGGTGAAAA

ΔCt [normalised ChIP] = (Ct [ChIP] – (Ct [Input] –Log2 (Input Dilution Factor).

Input Dilution Factor = (fraction of the input chromatin saved)–1 × Input dilution factor. We take 10 μL as input from 200 μL of IP sample and dilute input 10 times for RT-PCR. Input fraction (Input Dilution Factor) = 20 × 10 = 200.

ΔCt [normalised ChIP] = (Ct [ChIP] –(Ct [Input] – Log2 (200).

Finally, the percentage (Input %) value for each sample was calculated as Input % =100/2 ΔCt [normalised ChIP]. The “Input %” value represents the enrichment of certain histone modification in a specific region.

### Statistical analysis

Data are presented as means ± SD. A *t* test for paired data was used to determine the significance of the differences between two groups for the migration and invasion assay. Mann–Whitney test was used to determine the significance for the Western blot experiments. *P* < 0.05 was considered statistically significant.

## Results

### DAXX is downregulated in advanced gastric cancer

**Fig. 1:**
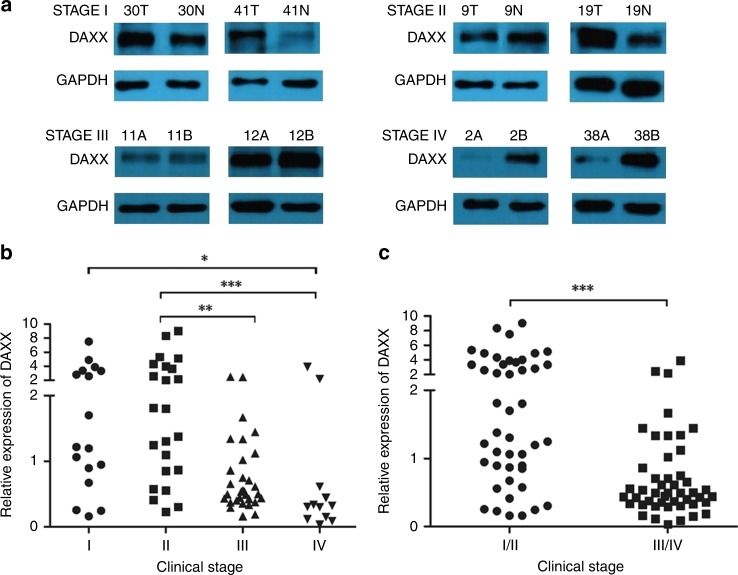
DAXX expression in gastric cancer primary samples from patients. **a** Representative western blots showing DAXX expression in stages I–IV GC samples and their matched adjacent non-neoplastic gastric tissues. 30T, 41T, 9T, 19T, 11A, 12A, 2A and 38A are clinical samples from patients with gastric cancer. 30N, 41N, 9N, 19N, 11B, 12B, 2B and 38B are matched adjacent gastric tissues. **b**, **c** DAXX relative expression in 83 gastric cancer samples. The bands were quantified by ImageJ and normalised to GAPDH. The normalised densities from gastric cancer samples were then divided by those of the corresponding adjacent gastric tissues. *P < 0.05, **P < 0.01, ***P < 0.001.

DAXX expression in clinical samples of different stages was examined by Western blot (Fig. 1). The total number of samples was 83, covering all 4 stages of GC, including 16 in stage I, 24 in stage II, 31 in stage III and 12 in stage IV. By comparing the ratio of DAXX expression between cancer and adjacent tissues in each stage, we found that DAXX expression in cancer tissues gradually decreased in more advanced stages of gastric cancer.

### Expression of DAXX correlates inversely with cancer stem cell markers

**Fig. 2:**
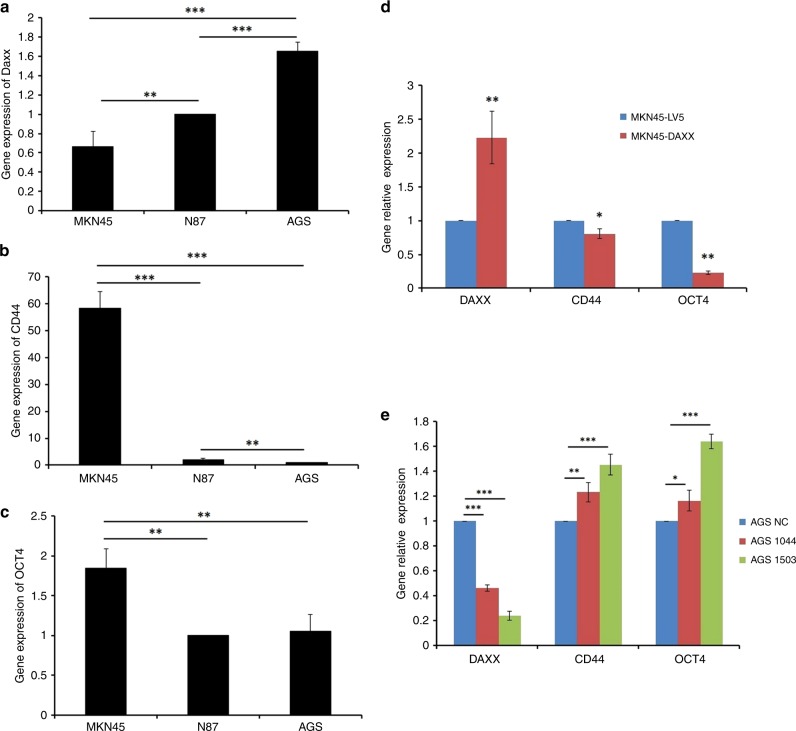
The expression of DAXX, CD44 and Oct4 in gastric cancer cell lines. **a**, **c** Real-time PCR analysis of DAXX, CD44 and OCT-4 mRNA levels in gastric cancer cell lines MKN45 (**a**), N87 (**b**) and AGS (**c**). **d**, **e** Real-time PCR analysis of DAXX, CD44 and OCT-4 mRNA levels in MKN45 cells transfected with a lentivirus that overexpresses DAXX (**d**), and in AGS cells transfected with a lentivirus that expresses two different DAXX shRNAs (**e**). *P < 0.05, **P < 0.01, ***P < 0.001.

**Fig. 3:**
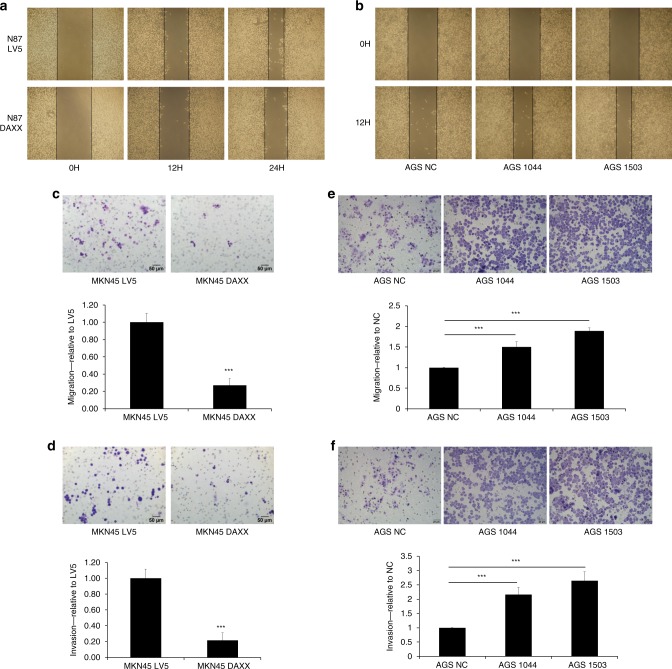
The effect of DAXX on migration and invasion of gastric cancer cells. **a**, **b** The effect of DAXX overexpression in N87 cells, and knockdown in AGS cells on migration by wound-healing assay. **c**, **d** Migration and invasion of MKN45 cells transfected with a lentivirus that overexpresses DAXX or vector alone (LV5). **e**, **f** Migration and invasion of AGS cells transfected with a lentivirus that expresses two different shRNAs or control (NC). ***P < 0.001.

CD44 and Oct4 are stem cell markers for gastric cancer cells.^7,8,16^ DAXX expression is the highest in AGS cells and the lowest in MKN45 cells (Fig. 2a), while the expression of CD44 and Oct4 is the highest in MKN45 cells (Fig. 2b, c). Considering the fact that tumorigenic potential of these three gastric cancer lines is in the order of MKN45 > N87 > AGS,^16^ our data suggest that DAXX may inhibit the growth of cancer stem cells. To verify this hypothesis, stable overexpression or knockdown of DAXX in MKN45 and AGS cells, respectively, was accomplished using lentiviral constructs. DAXX overexpression inhibited the expression of CD44 and Oct4, whereas DAXX knockdown increased the expression of these proteins (Fig. 2d, e). DAXX overexpression was associated with increased apoptosis (Supplementary Fig. 1), and decreased migration, wound healing and invasion (Fig. 3), whereas DAXX knockdown induced the opposite effects. DAXX overexpression in AGS cells inhibited the expression of anti-apoptotic genes, including *cIAP1, cIAP2, survivin, cFLIP, XIAP* and *Bcl-XL* (Supplementary Fig. 1).

It has been shown that the expression of CD44-high plus CD24-low combined demonstrates stem cell features in gastric cancer cells^16^ and breast cancer cells.^17^ Flow cytometry analysis showed that MKN45 cells contain a much higher percentage of CD44^high^/CD24^low^ cells compared with AGS cells. The expression of CD44^high^/CD24^low^ cells is decreased in MKN45 cells when DAXX was overexpressed (CD44 decreased from 87% to 61.43%; CD24 increased from 58.26 to 80%) (Supplementary Fig. 2A, B). In contrast, the expression of CD44^high^/CD24^low^ cells is increased in AGS cells when DAXX was silenced (CD44 increased from 68.4% to 92.8%; CD24 decreased from 98.2% to 88.8%) (Supplementary Fig. 2C, D). These findings were confirmed by Q-PCR (Supplementary Fig. 3A, B) and immunofluorescence staining (Supplementary Fig. 3C, D). We also analysed the expression of EMT markers by Q-PCR, and the results showed that DAXX overexpression resulted in increased mRNA levels of E-Cadherin, and decreased mRNA levels of Vimentin (Supplementary Fig. 3E). To confirm the inhibitory effect of DAXX on EMT marker expression, we repeated the experiment in AGS cells with DAXX knocked down. The result showed that DAXX silencing resulted in reduced expression of E-Cadherin and increased expression of Vimentin in AGS cells when DAXX was silenced (Supplementary Fig. 3F).

### HDAC-1 is recruited by DAXX to inhibit EMT

**Fig. 4:**
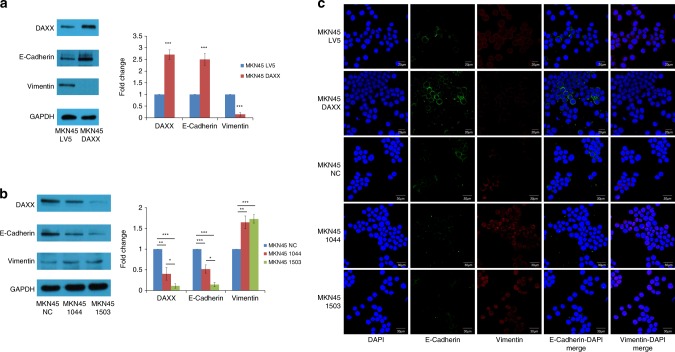
DAXX inhibits EMT of gastric cancer cells. **a** Western blot analysis of DAXX, E-cadherin and vimentin expression in MKN45 cells transfected with lentivirus overexpressing DAXX or vector control (LV5). **b** Western blot analysis of DAXX, E-cadherin and vimentin expression in MKN45 cells transfected with lentivirus that expresses two different DAXX shRNAs or vector control (NC). **c** Immunofluorescence staining of E-cadherin and vimentin in MKN45 cells transfected with lentivirus that overexpresses DAXX or expresses DAXX shRNA. *P < 0.05, **P < 0.01, ***P < 0.001.

In order to find out why DAXX overexpression or knockdown affects cell migration and invasion, we investigated possible involvement of DAXX in EMT, a biological process where epithelial cells lose cell-to-cell contact and transform to mesenchymal cells, and acquire their migratory and invasive properties. Western blotting and immunofluorescence results showed that EMT was inhibited in the MKN45 cells that overexpressed DAXX, characterised by upregulation of E-cadherin and downregulation of vimentin. In contrast, DAXX knockdown promoted EMT (Fig. 4).

AGS expresses high levels of DAXX; therefore, we knocked down DAXX in AGS and analysed the expression of E-Cadherin and Vimentin by immunostaining. The results showed that DAXX knockdown resulted in decreased expression of E-Cadherin and increased expression of Vimentin (Supplementary Fig. 4). These results confirmed the inhibitory effect of DAXX on EMT.

To provide morphological evidence of EMT, we performed DAXX overexpression and knockdown experiments using MKN45 and AGS cells, respectively, and then observed the effect of DAXX manipulation on EMT. MKN45 cells convert from an elongated mesenchymal shape to “cuboidal” epithelial structure when DAXX was overexpressed (Supplementary Fig. 5A). In contrast, AGS cells convert from a “cuboidal” epithelial structure into an elongated mesenchymal shape when DAXX was silenced (Supplementary Fig. 5B).

Figure 4a shows that MKN45-LV5 cells co-express E-cadherin and Vimentin at least at a population level. This is representative of partial EMT, which exhibits a mix of epithelial and mesenchymal traits, such as co-expression of epithelial (E-cadherin) and mesenchymal (Vimentin) markers. Partial EMT in cancer cells is thought to enhance their invasive properties, generate cancer stem cells, promote resistance to anticancer drugs and a poor prognosis.^18^ Indeed, we found more hybrid E/M cells in N87 cells with DAXX silencing (Supplementary Fig. 6).

**Fig. 5:**
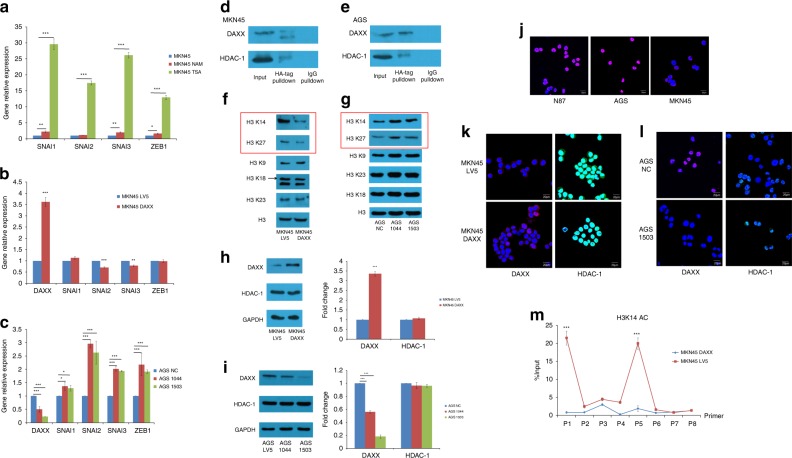
DAXX inhibits SNAI3 transcription by recruiting HDAC-1 into the nucleus. **a** Real-time PCR analysis of *SNAI* and *ZEB1* mRNA levels in MKN45 cells treated with NAM and TSA. **b**, **c** Real-time PCR analysis of *SNAI* and *ZEB1* mRNA levels in MKN45 cells transfected with lentivirus that overexpresses DAXX (**b**), and in AGS cells transfected with lentivirus that expresses two different DAXX shRNAs (**c**). **d**, **e** Pulldown of DAXX and HDAC-1 from MKN45 (**d**) and AGS (**e**) cells by a rabbit anti-HA-tag antibody or an isotype-matched control antibody. **f** Western blot analysis of H3K14ac, H3K27ac, H3K9ac, H3K18ac and H3K23ac expression in MKN45 cells transfected with lentivirus overexpressing DAXX or vector control. **g** Western blot analysis of H3K14ac, H3K27ac, H3K9ac, H3K18ac and H3K23ac expression in AGS cells transfected with lentivirus that expresses DAXX shRNA or vector control. **h** Western blot analysis of DAXX and HDAC-1 expression in MKN45 cells transfected with lentivirus overexpressing DAXX and vector control. **i** Western blot analysis of DAXX and HDAC-1 expression in AGS cells transfected with lentivirus that expresses DAXX shRNA or vector control. **j** Localisation of DAXX expression in MKN45, N87 and AGS cells by immunofluorescence staining. **k** Localisation of DAXX and HDAC-1 in MKN45 cells transfected with a lentivirus that overexpresses DAXX or vector control. DAXX (red), HDAC-1 (green) and DAPI (blue). **l** Localisation of DAXX and HDAC-1 expression in AGS cells transfected with a lentivirus that expresses DAXX shRNA or vector control. **m** The enrichment of SNAI3 promoter region on H3K14ac in MKN45 cells transfected with lentivirus overexpressing DAXX or vector control. *P < 0.05, **P < 0.01, ***P < 0.001.

A recent study reported that DAXX inhibits EMT by preventing the interaction between Slug (SNAI2) and the histone deacetylase HDAC-1.^9^ To explore if DAXX’s inhibitory effect on EMT and stemness could be mediated by histone deacetylase, we detected the expression of CD44, OCT-4, TWIST, SNAI and ZEB after MKN45 was treated with nicotinamide (NAM) and trichostatin A (TSA), inhibitors of sirtuin and HDAC, respectively. The expression of SNAI and ZEB increased greatly after TSA treatment (Fig. 5a), suggesting potential involvement of HDAC in regulating EMT. We further examined the effects of DAXX modulation on SNAI and ZEB mRNA expression. SNAI2 and SNAI3 mRNA levels were reduced when DAXX was overexpressed, but increased when DAXX was knocked down, suggesting that DAXX could repress EMT by inhibiting the transcription of SNAI2 and SNAI3 (Fig. 5b, c).

HDAC-1 is involved in the transcriptional regulation of multiple proteins. It has been shown that HDAC-1 represses the transcription of SNAI2 in lung cancer cells.^19^ However, the interactions between DAXX and HDAC-1 have not been reported. To examine whether DAXX could bind to HDAC-1, we performed immunoprecipitation using HA antibody, followed by Western blotting, using antibodies against DAXX or HDAC-1. Both DAXX and HDAC-1 were detected in the proteins pulled down by the HA antibody in gastric cancer cells overexpressing DAXX (Fig. 5d, e), suggesting that DAXX and HDAC-1 are physically associated within these cells. We further showed that both DAXX and HDAC-1 migrated into the nucleus when DAXX was overexpressed in MKN45 cells (Fig. 5k), while HDAC-1 underwent partial cytoplasmic migration when DAXX was knocked down in AGS cells (Fig. 5l), implying that HDAC-1 can be recruited into the nucleus when DAXX is overexpressed. Our findings are in agreement with a report by Zhong et al., showing that at steady state, DAXX was localised to the cytosol of splenocytes, but when DAXX expression was induced by Con A, DAXX was mainly localised to the nucleus.^20^ When Cos-1 cells were transiently transfected with vector expressing DAXX, its localisation was also found in the nucleus.^20^ In agreement with these findings, our data also showed that wild-type gastric cancer lines, AGS, N87 and MKN45, express different levels of DAXX. DAXX expression is the highest in AGS cells and the lowest in MKN45 cells (Fig. 2a). Immunofluorescence results revealed that DAXX was mainly localised in the nucleus in both N87 and AGS cells, whereas it was mostly present in the cytoplasm in MKN45 cells (Fig. 5j).

To demonstrate how HDAC-1 is recruited to the nucleus, we immunoprecipitated endogenous HDAC-1 or DAXX using rabbit anti-HDAC-1 antibody or rabbit anti-DAXX antibody from AGS cell nuclear lysates. The immunoprecipitates were then analysed by Western blot using antibodies against DAXX or HDAC-1 (Supplementary Fig. 7). The results showed that the endogenous DAXX protein associated with HDAC-1 in vivo.

It was shown that DAXX can be re-localised to the cytoplasm during oxidative stress induced by either glucose deprivation or H_2_O_2_ treatment.^21^ After 48 h of incubation, AGS cells were exposed to glucose-free medium for 1 h or H_2_O_2_ (500 µM) for 30 min. Immunofluorescent staining revealed that both DAXX and HDAC-1 moved to the cytoplasm (Supplementary Fig. 7C). These results indicate that DAXX and HDAC-1 are physically associated, and that HDAC-1 can be recruited to the nucleus by DAXX through direct physical association.

To determine whether DAXX affects the acetylation level of gastric cancer cells, we detected the changes in acetylation level of H3 acetylation sites after DAXX overexpression and knockdown by Western blotting. The acetylation levels of H3 acetylation sites in H3K14 and H3K27 were reduced when DAXX was overexpressed, and increased when DAXX was knocked down (Fig. 5f, g).

DAXX overexpression or knockdown did not alter the expression level of HDAC-1 as shown by Western blotting (Fig. 5h, i). However, immunofluorescence results revealed that DAXX was mainly localised in the nucleus in both N87 and AGS cells, whereas it was mostly present in the cytoplasm in MKN45 cells (Fig. 5j). Both DAXX and HDAC-1 migrated into the nucleus when DAXX was overexpressed in MKN45 cells (Fig. 5k), while HDAC-1 underwent partial cytoplasmic migration when DAXX was knocked down in AGS cells (Fig. 5l), implying that HDAC-1 can be recruited into the nucleus when DAXX is overexpressed.

To determine whether H3K14 acetylation can affect SNAI3 transcription, we performed ChIP assay in MKN45 cells overexpressing DAXX. We designed a nest of primers, 250 bp each, covering 2000 bp upstream of SNAI3 starting codon ATG, and used ChIP products as templates for ChIP-qPCR. The results showed that the two sequences of 832–1110 bp and 1972–2267 bp upstream of SNAI3 are enriched on H3K14ac in MKN45 cells, and the enrichment was reduced when DAXX was overexpressed (Fig. 5m), suggesting that DAXX inhibits the expression of SNAI3 by downregulating the acetylation level of H3K14 in gastric cancer cells. To confirm that the effect of DAXX on SNAI3, EMT and stemness is through HDAC-1, we performed rescue experiments to show how the effects of DAXX change when cells are treated with HDAC inhibitors. We analysed the expression of SNAI3, EMT and stem cell markers in MKN45 cells transfected with lentivirus overexpressing DAXX in the presence or absence of HDAC inhibitor TSA. Western blot and real-time PCR analysis showed that the increased expression of E-Cadherin and decreased expression of Vimentin resulting from DAXX overexpression was reversed by TSA (Supplementary Fig. 8A-B). To verify these findings, we transfected AGS cells with lentivirus carrying shRNA-targeting DAXX in the presence or absence of HDAC inhibitor TSA. Western blot and real-time PCR analysis showed that the decreased expression of E-Cadherin and increased expression of Vimentin resulting from DAXX silencing was reversed by TSA (Supplementary Fig. 8C-D). Real-time PCR analysis also showed that the decreased expression of SNAI3 resulting from DAXX overexpression, and the increased expression of SNAI3 resulting from DAXX silencing, were reversed by TSA (Supplementary Fig. 8E-H).

GRHL2 and OVOL2 are two important transcription factors that inhibit EMT, as described by Mooney et al.^22^ We analysed the expression of GRHL2 and OVOL2 in MKN45 cells transfected with lentivirus overexpressing DAXX. Real-time PCR analysis showed that the expression of GRHL2 and OVOL2 is increased when DAXX was overexpressed (Supplementary Fig. 9A). To verify these findings, we transfected AGS cells with lentivirus carrying lentivirus-expressing shRNA-targeting DAXX. Real-time PCR analysis showed that the expression of GRHL2 and OVOL2 was decreased when DAXX was knocked down (Supplementary Fig. 9B).

### Knockdown of DAXX enhances anchorage-independent growth and drug resistance in gastric cancer cells

**Fig. 6:**
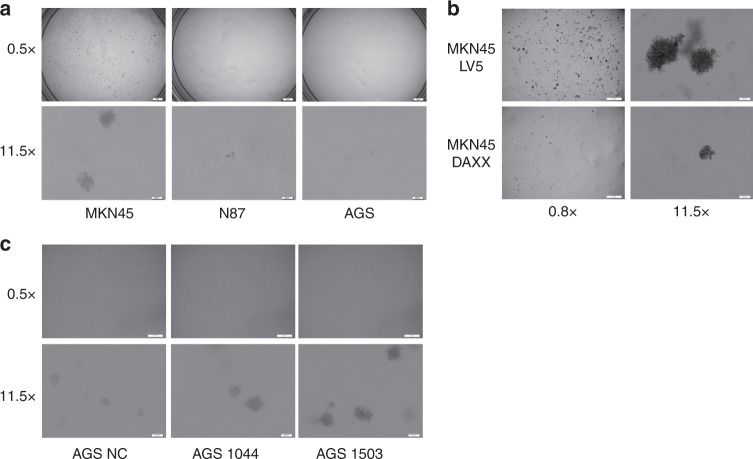
Effect of DAXX on anchorage-independent growth of gastric cancer cells. **a** Soft agar colony-formation assay of gastric cancer cells (10 days). **b** The effect of DAXX overexpression on the ability of MKN45 cells to form colonies. **c** Soft agar colony assay of AGS cells transfected with lentivirus that expresses two different DAXX shRNAs or vector control (25 days).

Soft agar assay demonstrated that MKN45 cells formed more colonies that were also larger in size than those observed in N87 and AGS cells (Fig. 6a), which is consistent with the tumorigenic potential of these cell lines.^7,8,16^ The colony-formation potential of MKN45 cells was significantly reduced when DAXX was overexpressed, whereas it was enhanced when DAXX was knocked down (Fig. 6b, c), suggesting that DAXX inhibits anchorage-independent growth of gastric cancer cells.

To assess whether DAXX affects cancer cell resistance to chemotherapy, we treated the cells with 5-fluorouracil (5-FU) and etoposide, two commonly used chemotherapeutics for gastric cancer. Cell viability assay revealed that MKN45 cells were much more resistant to the treatment than N87 and AGS cells (Supplementary Fig. 10A, B). However, MKN45 cells became more sensitive to the drug treatment when DAXX was overexpressed. In contrast, AGS cells became more resistant to drug treatment when DAXX was knocked down (Supplementary Fig. 10C–F).

### Overexpression of DAXX inhibits tumour formation in vivo

To assess the role of DAXX in tumour development in vivo, we injected MKN45 cells transfected with a lentivirus that overexpresses DAXX, or lentivirus that carries vector alone in nude mice. DAXX overexpression significantly inhibited tumour growth as documented by the decrease in tumour size and weight (Supplementary Fig. 11 A–D). IHC assay of CD44 expression in frozen sections from mouse tumour revealed that tumour stemness decreased in tumours overexpressing DAXX (Supplementary Fig. 11E). To confirm these findings, we injected AGS cells transfected with a lentivirus that expresses DAXX shRNA. Wild-type AGS cells failed to form tumours in nude mice, which is consistent with previous reports that AGS cells do not produce spheroid colonies in vitro, and lack the ability to form tumours in SCID mice.^16^ However, AGS cells transfected with lentivirus that expresses shRNA-1503 but not 1044 were able to form a small but visible tumour, which was consistent with their knockdown efficiency (Supplementary Fig. 11F–H).

## Discussion

DAXX is a transcription repressor involved in the regulation of cell proliferation, apoptosis and the development of cancer. Our study demonstrates for the first time that DAXX inhibits the growth of gastric cancer by suppressing EMT. In agreement with this notion, overexpression of DAXX in MKN45 cells inhibited EMT by downregulating SNAI3 transcription. The mechanisms by which DAXX induces downregulation of SNAI3 in gastric cancer cells are not clear. It has been shown that HDAC-1 represses the transcription of SNAI2 in lung cancer cells.^19^ Indeed, our immunofluorescence data revealed that DAXX overexpression in MKN45 cells led to translocation of HDAC-1 from the cytoplasm to the nucleus, suggesting that DAXX overexpression-induced downregulation of SNAI3 could be mediated by HDAC-1.

A recent study reported that DAXX could bind to the DNA-binding domain of SNAI2, and prevent its interaction with HDAC-1, which is required for the repression of E-cadherin transcription and initiation of EMT.^9^ Based on our data that DAXX forms a complex with HDAC-1, and that DAXX causes the migration of HDAC-1 into the nucleus, we propose a model in which DAXX inhibits EMT by recruiting HDAC-1 into the nucleus, and represses the transcription of *SNAI3*.

In this study, we identified a novel inverse correlation between DAXX expression and that of the cancer stem cell markers CD44 and Oct4. We further showed that DAXX inhibited anchorage-independent growth of gastric cancer cells, which became more sensitive to chemotherapies when DAXX was overexpressed. With regard to the role of DAXX in the regulation of stem cell differentiation, we only found two reports in the literature—DAXX inhibits muscle stem cell differentiation by repressing E2A-dependent expression of key myogenic genes via HDAC recruitment to E2A-dependent promoters,^23^ as well as DAXX is involved in cell fate conversions through transcriptomic rewiring by regulating the deposition of H3.3 on heterochromatin.^24^ Although we do not know how DAXX regulates the expression of CD44 and Oct4, it is likely that DAXX-mediated epigenetic modifications play a role.

Taken together, our data provide new insights into the role of DAXX as a tumour suppressor in gastric cancer, and suggest that upregulation of DAXX can overcome chemoresistance. These findings might open a new door for the development of novel targeted therapy for gastric cancer that depends on DAXX exploitation.

## Supplementary information

Supplementary Figures

## Data Availability

All pertinent data to support this study are included in the paper and supplementary files. Further data supporting the findings are available upon request.
