# Supplementary material for: DAXX inhibits cancer stemness and epithelial–mesenchymal transition in gastric cancer
Source: Br J Cancer. 2020 Mar 23;122(10):1477–85. doi: 10.1038/s41416-020-0800-3 (PMC7217831; doi:10.1038/s41416-020-0800-3)
Supplement: Supplementary file 1 — Supplementary Figures [file 41416_2020_800_MOESM1_ESM.doc]

**
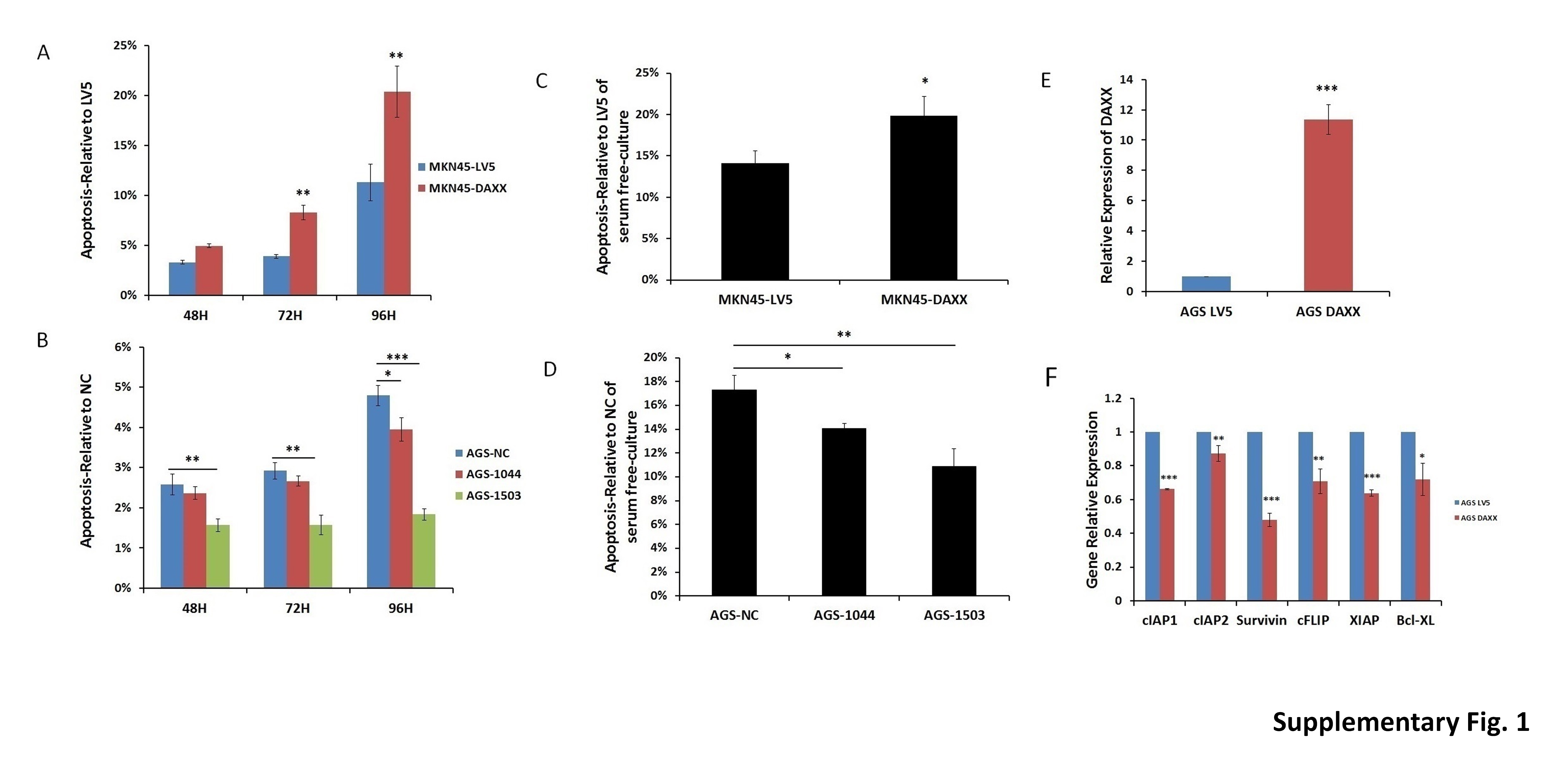
**

**Supplementary Fig. 1. The effect of DAXX on apoptosis of gastric cancer cells.** (A) Percentage of apoptosis of MKN45 cells transfected with a lentivirus that overexpresses DAXX or vector alone (LV5). (B) Percentage of apoptosis of AGS cells transfected with a lentivirus that expresses two different shRNA or control (NC). (C) Percentage of apoptosis of MKN45 cells transfected with a lentivirus that overexpresses DAXX or vector alone (LV5) in serum-free condition. (D) Percentage of apoptosis of AGS cells transfected with a lentivirus that expresses two different shRNA or control (NC) in serum-free condition. (E) Real-time PCR analysis of DAXX mRNA level in AGS cells transfected with a lentivirus that overexpresses DAXX or vector alone (LV5). (F) Real-time PCR analysis of anti-apoptotic genes in AGS cells transfected with a lentivirus that overexpresses DAXX or vector alone (LV5). *P < 0.05, **P < 0.01, ***P < 0.001


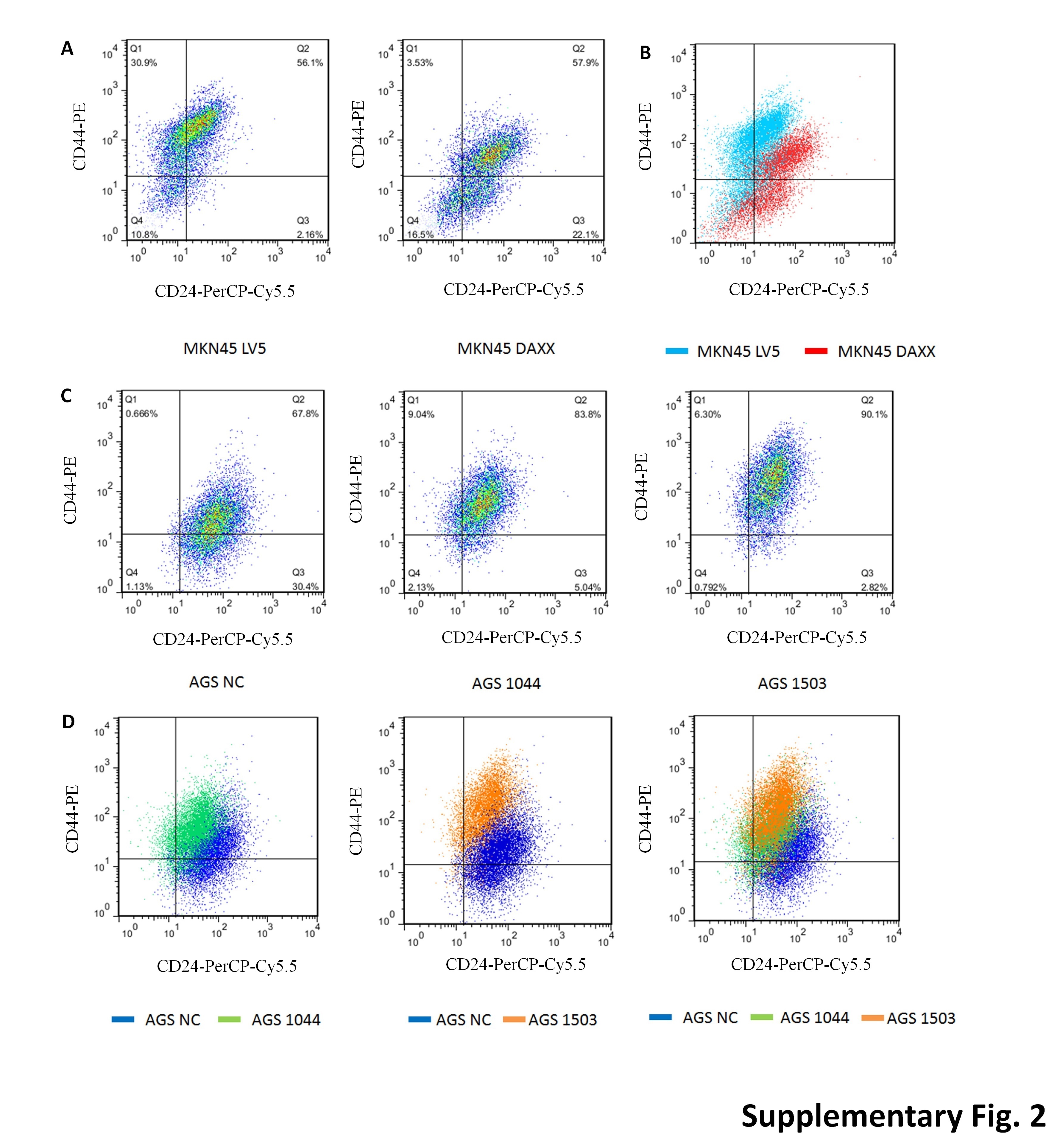


**Supplementary Fig. 2. The effect of DAXX on CD44 and CD24.** (A) Flow cytometry analysis CD44 and CD24 expression in MKN45 cells transfected with lentivirus overexpressing DAXX and vector control. (B) MKN45-LV5 (blue) and MKN45-DAXX (red) merge. (C) Flow cytometry analysis CD44 and CD24 expression in in AGS cells transfected with DAXX shRNA (1044 and 1503) and vector control. (D) AGS-NC (blue), AGS-1044 (green) and AGS-1503 (orange) merge.


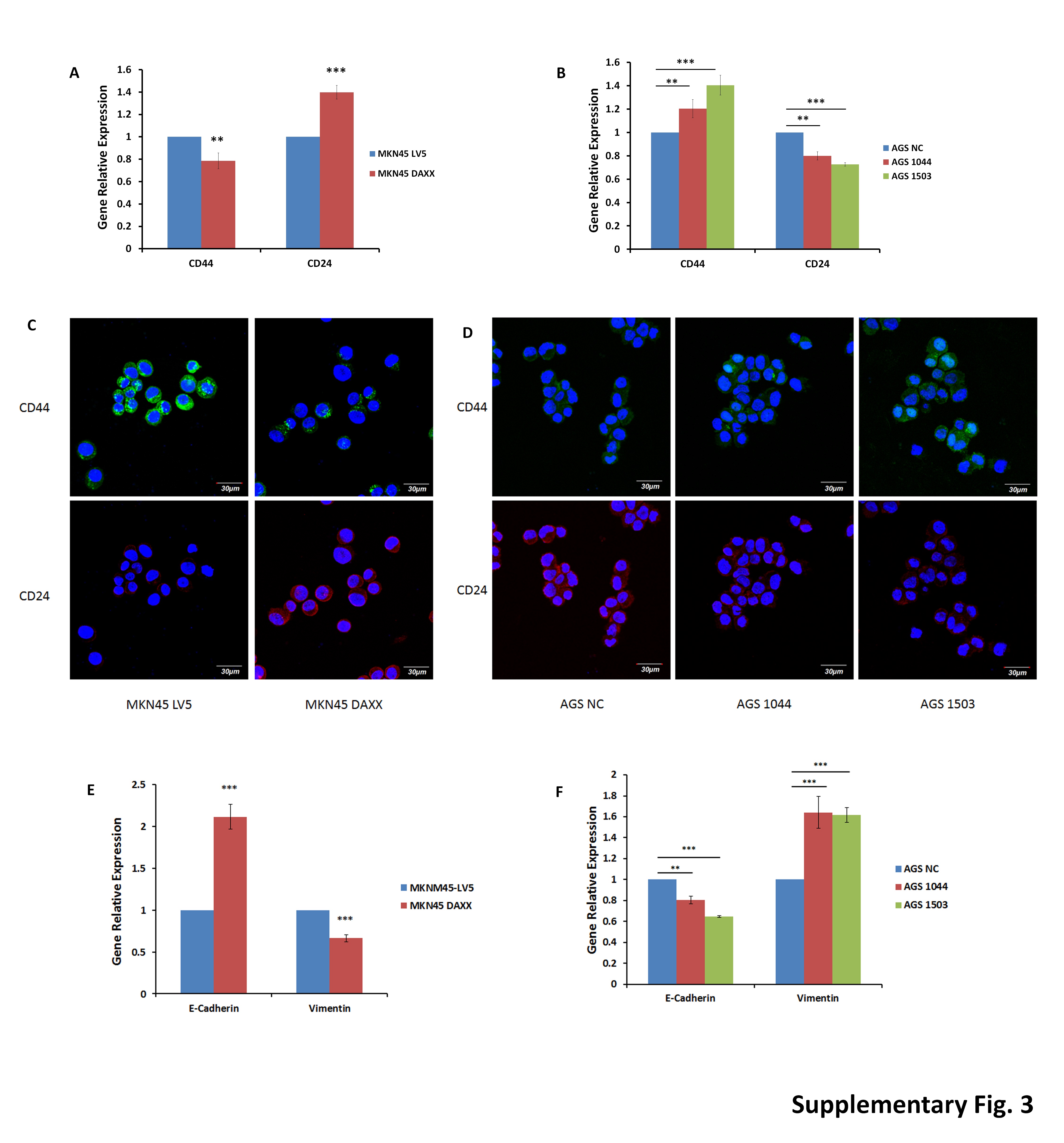


**Supplementary Fig. 3. The effect of DAXX on CD44 and CD24.** (A-B) Q-PCR analysis of CD44 and CD24 expression in MKN45 cells transfected with lentivirus overexpressing DAXX and vector control (A) or AGS cells transfected with DAXX shRNA (1044 and 1503) and vector control (B). (C-D) Immunofluorescence analysis of CD44 and CD24 in MKN45 cells transfected with lentivirus overexpressing DAXX and vector control (C) or AGS cells transfected with DAXX shRNA (1044 and 1503) and vector control (D). (E-F) Q-PCR analysis of E-Cadherin and Vimentin expression in MKN45 cells transfected with lentivirus overexpressing DAXX and vector control (E) or AGS cells transfected with DAXX shRNA (1044 and 1503) and vector control (F). ** p<0.01, *** p<0.001


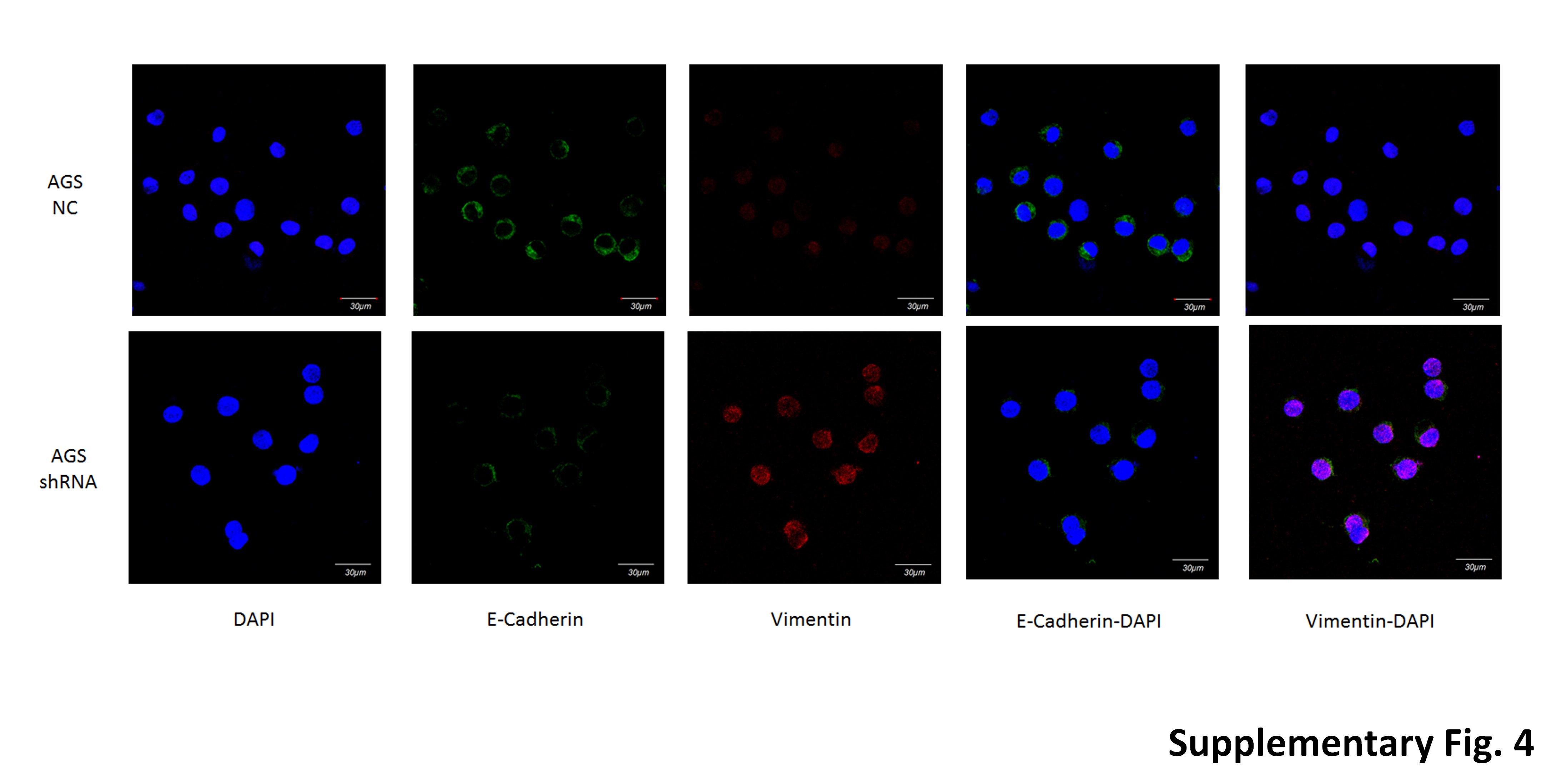


**Supplementary Fig. 4. The effect of DAXX knock down on EMT in AGS.** Immunofluorescence analysis of E-Cadherin and Vimentin in AGS cells transfected with DAXX shRNA and vector control.


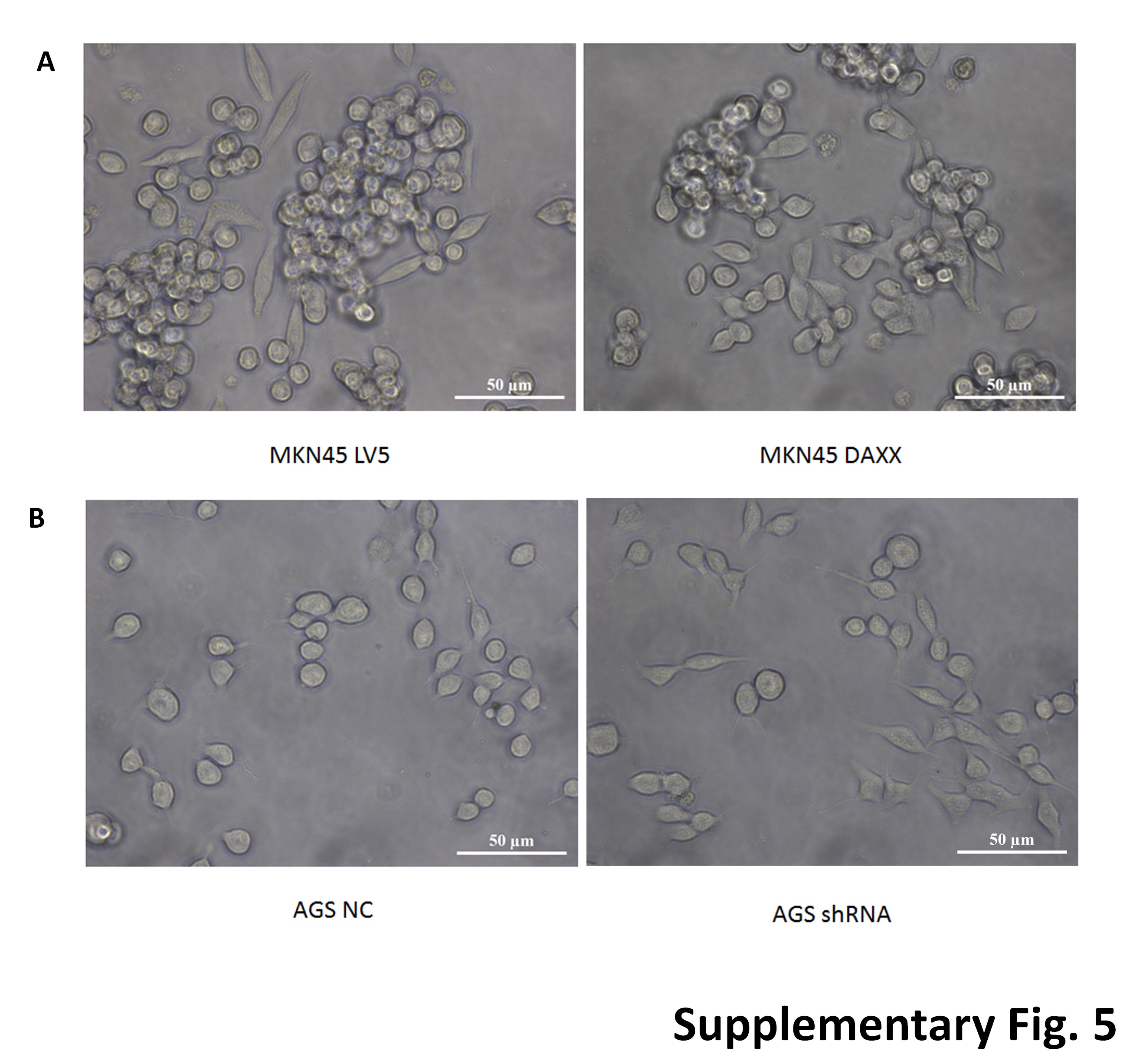


**Supplementary Fig. 5. The effect of DAXX on cell morphology.** Cells observed by inverted microscope. (A) MKN45 cells transfected with lentivirus overexpressing DAXX and vector control. (B) AGS cells transfected with DAXX shRNA and vector control.


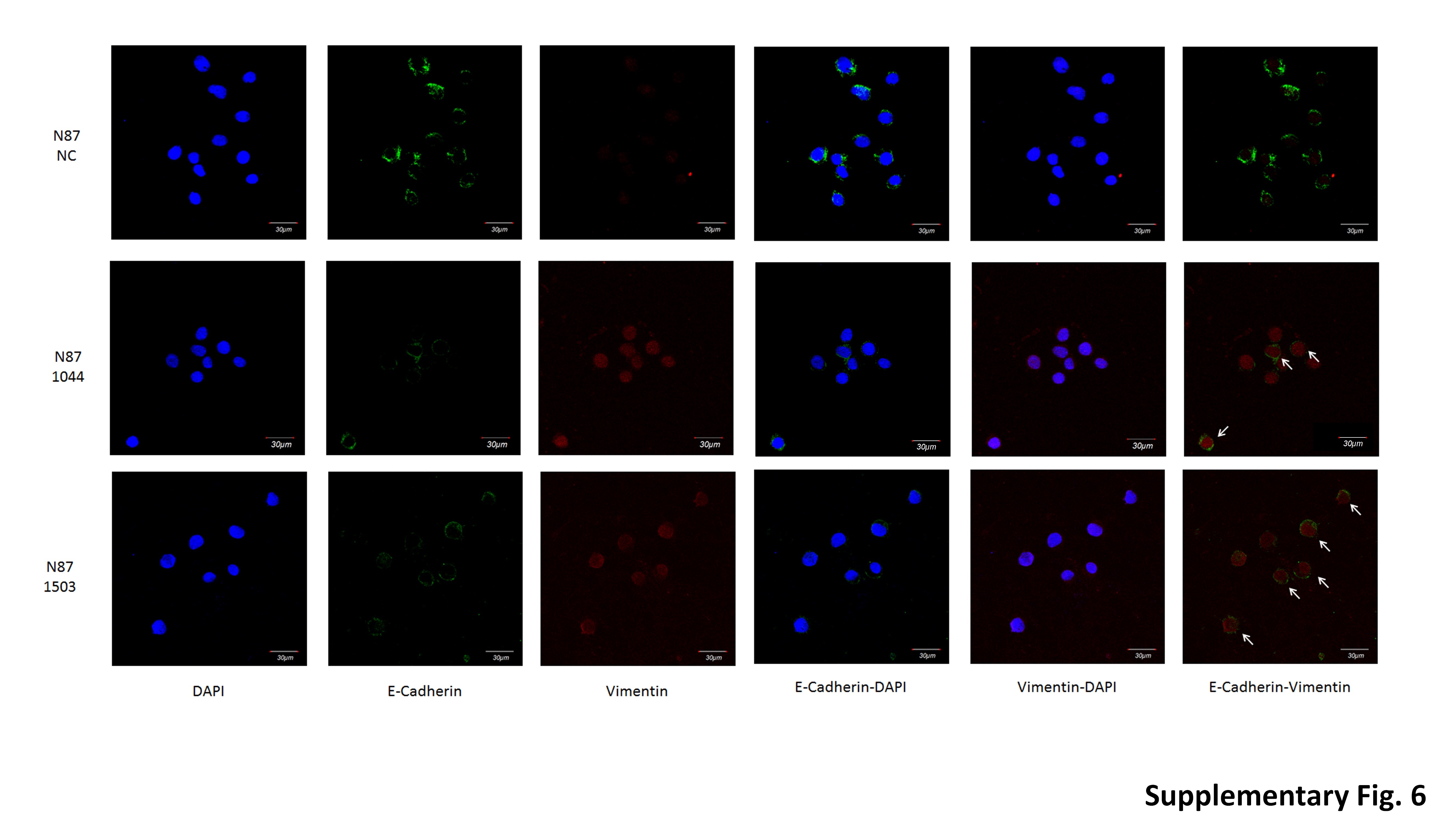


**Supplementary Fig. 6. The effect of DAXX on hybrid E/M cells.** Immunofluorescence analysis of E-Cadherin and Vimentin in N87 cells transfected with DAXX shRNA (1044 and 1503) and vector control. The white arrow shows the hybrid E/M cells.


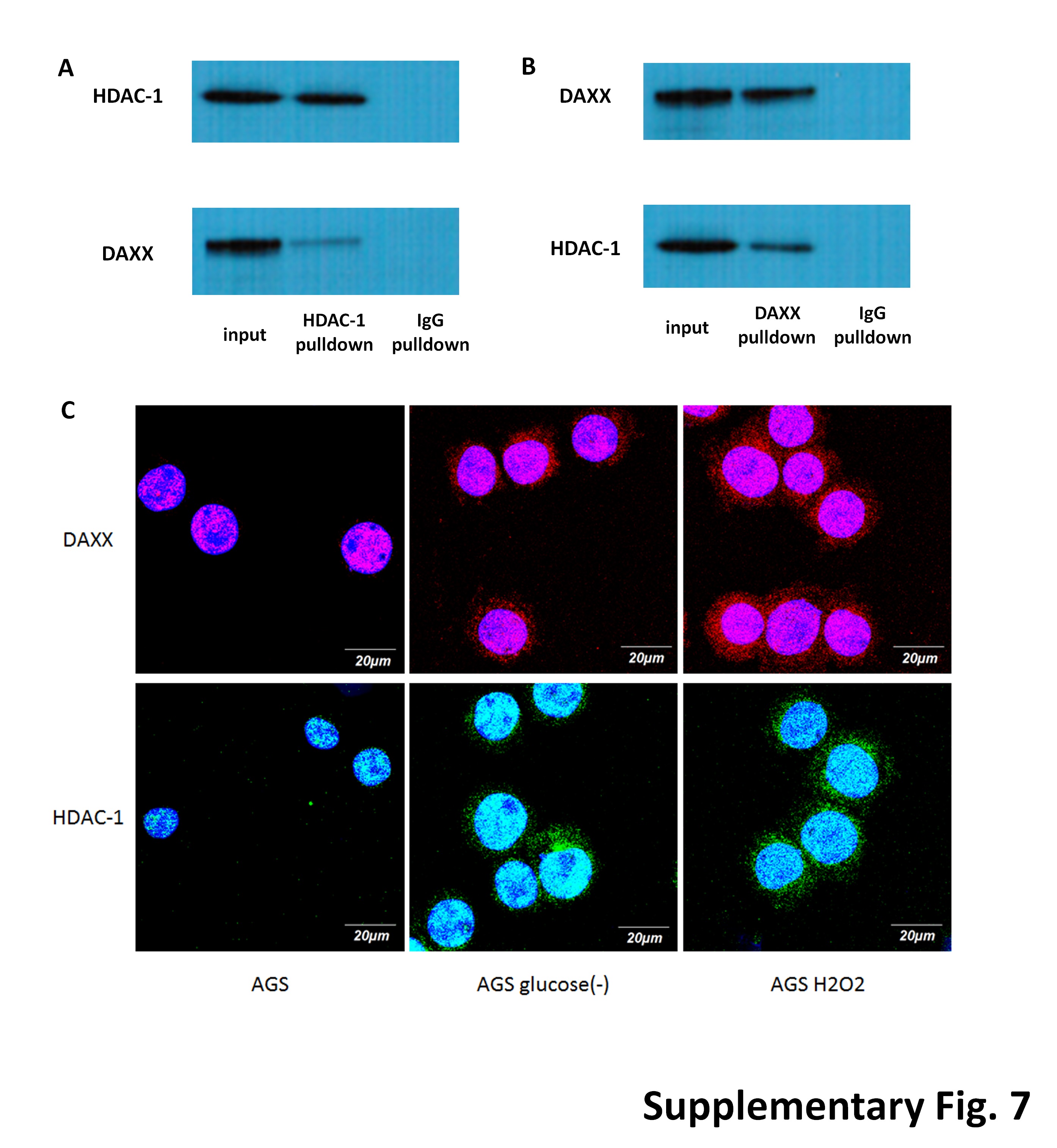


**Supplementary Fig. 7. HDAC-1 is recruited to nucleus by DAXX.** (A-B) Protein extracted from the nuclear of AGS cells were immunoprecipitated by rabbit anti-HDAC-1 antibodies (A) or rabbit anti-DAXX (B) or isotype-matched control antibodies. Western blot analysis of DAXX and HDAC-1 expression in proteins which has been pulled down. (C) Immunofluorescence analysis the localization of DAXX in AGS cells after exposed to glucose free medium for 1 h or H2O2 (500 uM) for 30 min.


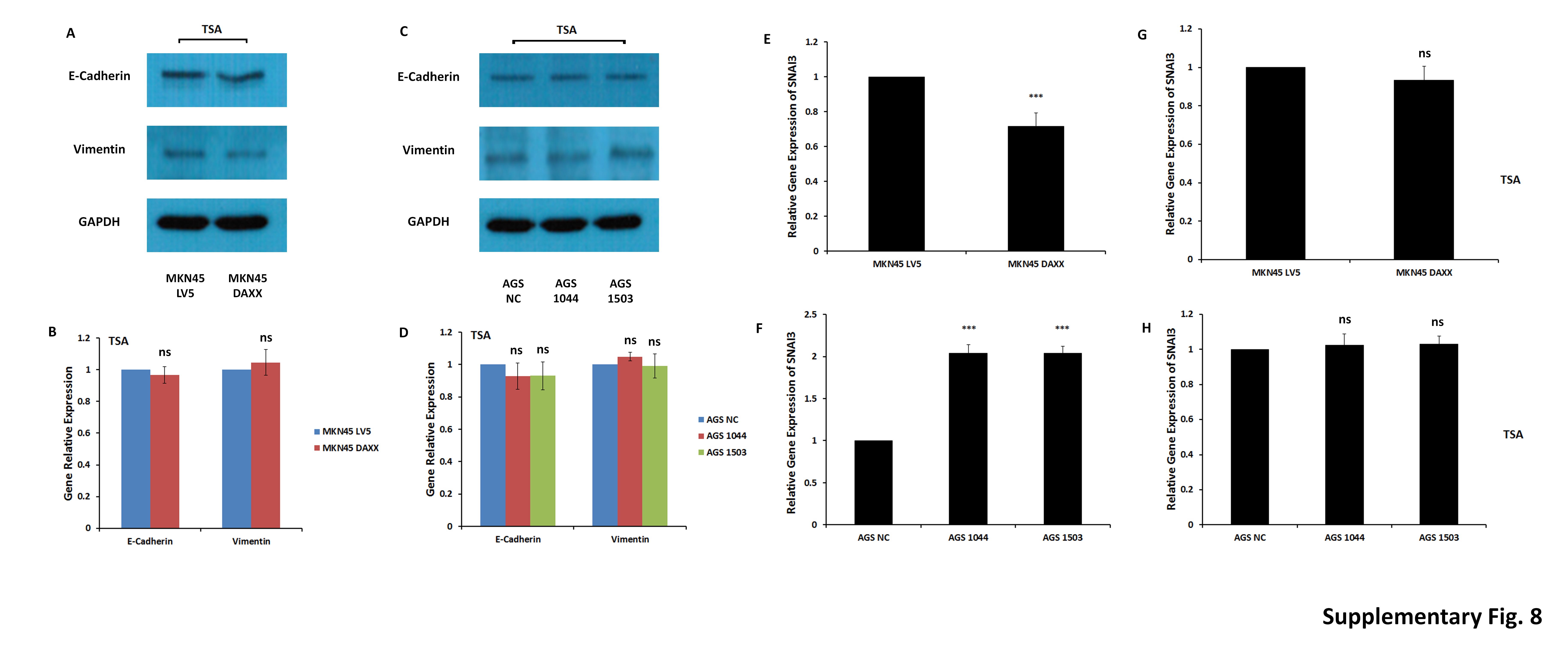


**Supplementary Fig. 8. The effects of DAXX were reversed by TSA.** (A-B) Western blot (A) and q-PCR (B) analysis of E-Cadherin and Vimentin expression in MKN45 cells transfected with lentivirus overexpressing DAXX and vector control treated with 1uM TSA for 6 hours. (C-D) Western blot (C) and q-PCR (D) analysis of E-Cadherin and Vimentin expression in AGS cells transfected with DAXX shRNA (1044 and 1503) and vector control treated with 1uM TSA for 6 hours. (E-F) Q-PCR analysis of SNAI3 expression in MKN45 cells transfected with lentivirus overexpressing DAXX and vector control (E) or AGS cells transfected with DAXX shRNA (1044 and 1503) and vector control (F). (G-H) Q-PCR analysis of SNAI3 expression in MKN45 cells transfected with lentivirus overexpressing DAXX and vector control (G) or AGS cells transfected with DAXX shRNA (1044 and 1503) and vector control (H) treated with 1uM TSA for 6 hours.


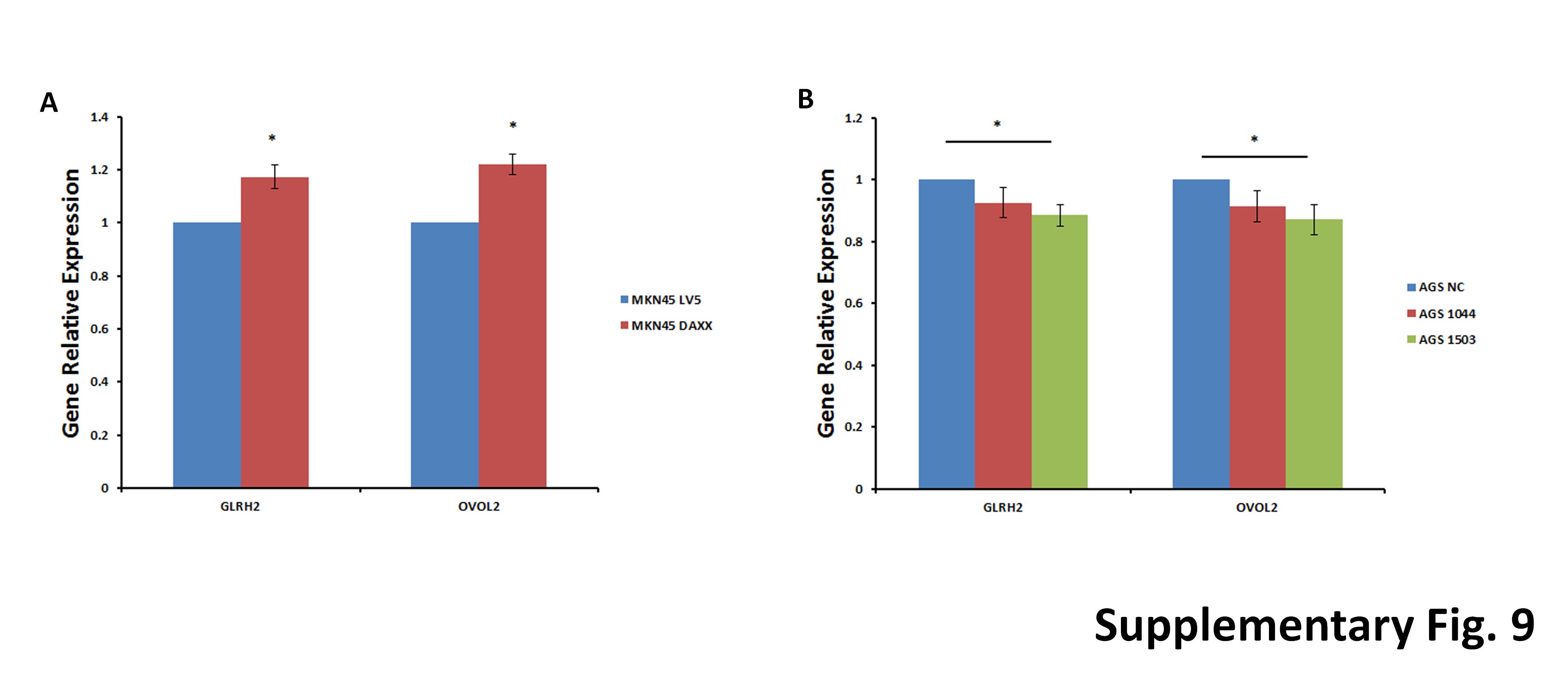


**Supplementary Fig. 9. The effect of DAXX on EMT-inhibiting TFs.** (A) Q-PCR analysis of GRHL2 and OVOL2 expression in MKN45 cells transfected with lentivirus overexpressing DAXX and vector control. (B) Q-PCR analysis of GRHL2 and OVOL2 expression in AGS cells transfected with DAXX shRNA (1044 and 1503) and vector control. * p<0.05


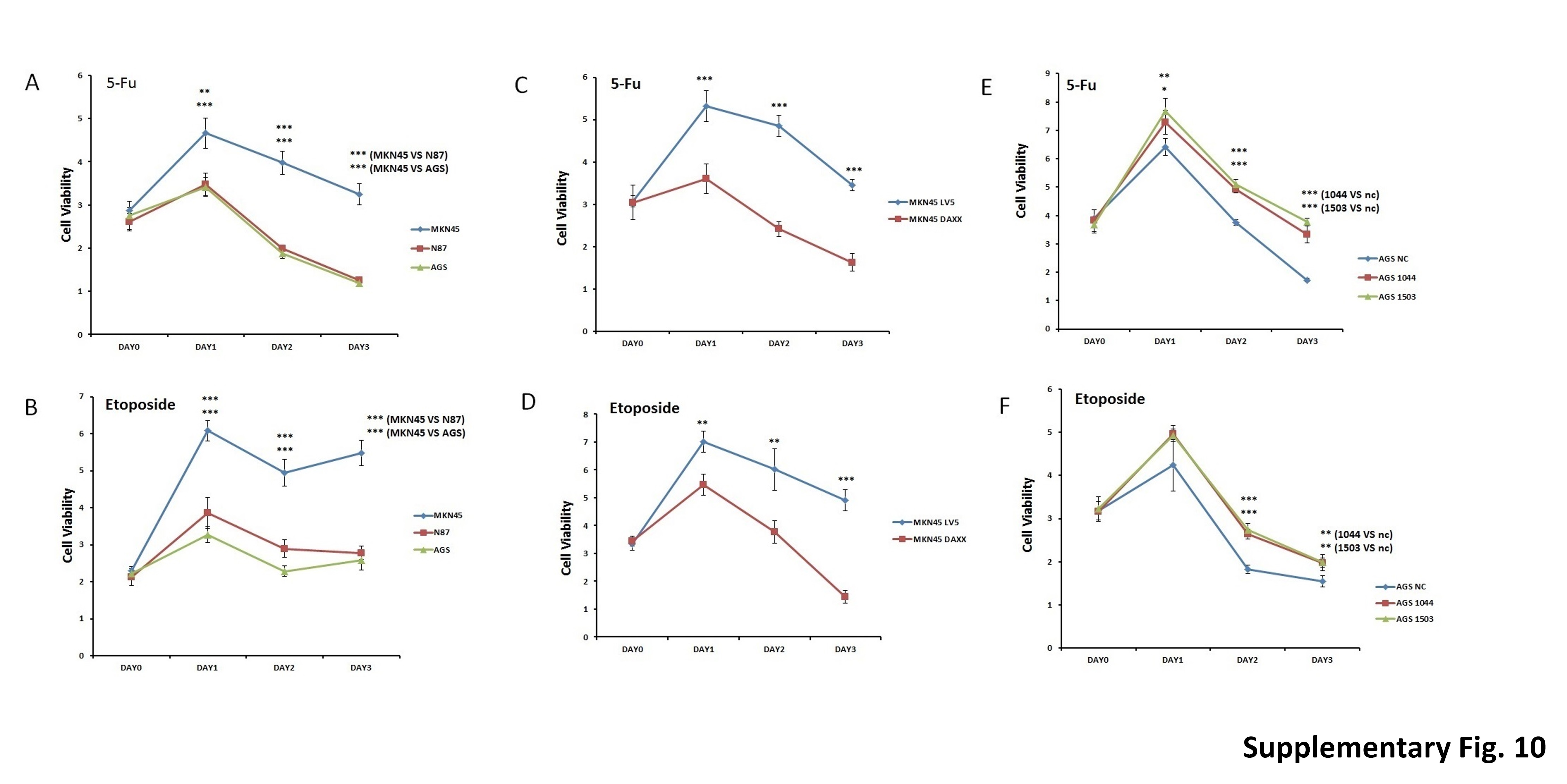


**Supplementary Fig. 10. The effect of DAXX on chemoresistance.** Cell viability was measured after 5-Fu and etoposide treatment. (A) MKN45, N87, and AGS cells were treated with 4 mM 5-Fu. (B) MKN45, N87, and AGS cells were treated with 80 uM etoposide. (C) MKN45 cells transfected with lentivirus overexpressing DAXX or vector control were treated with 2.5 mM 5-FU. (D) MKN45 cells transfected with lentivirus overexpressing DAXX or vector control were treated with 50 uM etoposide. (E) AGS cells transfected with DAXX shRNA or vector control were treated with 4 mM 5-FU. (F) AGS cells transfected with lentivirus that expresses two different DAXX shRNA or vector control were treated with 80 uM etoposide. *P < 0.05, **P < 0.01, ***P < 0.001


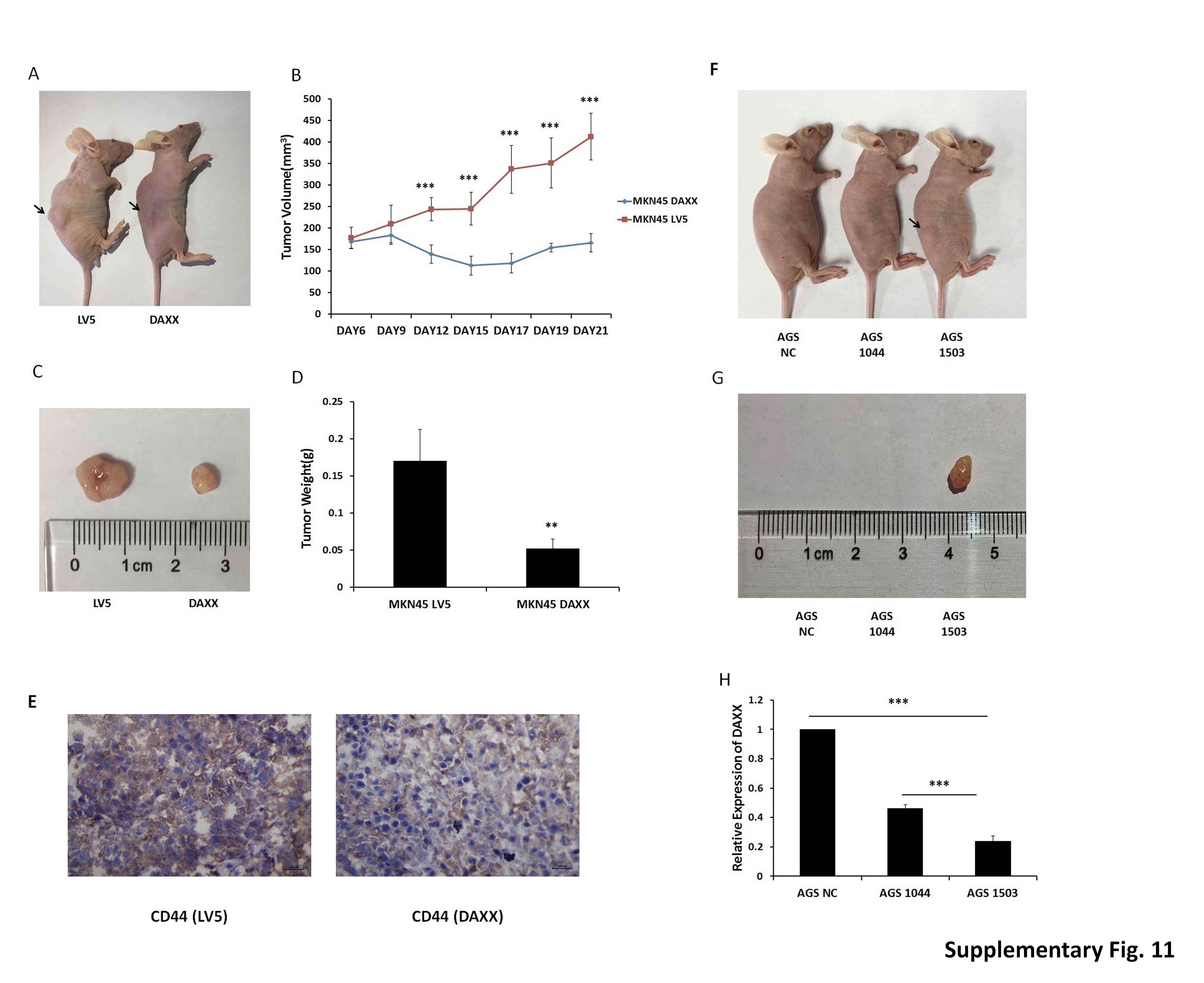


**Supplementary Fig. 11. DAXX inhibits gastric cancer growth *in vivo*.** **(A-D)** 8 x 10^6^ MKN45 transfected with lentivirus overexpressing DAXX or vector control were injected into nude mice subcutaneously (n=6/group). Tumors were harvested at 21 days after injection. **(E)** Immunohistochemical staining of CD44 (brown) in tumor transfected with lentivirus that overexpresses DAXX or control. **(F-G)** 1 x 10^7^ AGS cells transfected with lentivirus that expresses DAXX shRNA or vector control were injected into nude mice subcutaneously (n=6/group). Tumors were harvested at 35 days after injection. **(H)** Real-time PCR analysis of *DAXX* mRNA levels in AGS cells transfected with lentivirus that expresses two different DAXX shRNA or vector control. **P < 0.01, ***P < 0.001.
